# On the role of DNA biomechanics in the regulation of gene expression

**DOI:** 10.1098/rsif.2011.0371

**Published:** 2011-08-24

**Authors:** J. N. Milstein, J.-C. Meiners

**Affiliations:** Departments of Physics and Biophysics, University of Michigan, Ann Arbor, MI 48109, USA

**Keywords:** DNA mechanics, mechanoregulation, mechanome, mechanotransduction

## Abstract

DNA is traditionally seen as a linear sequence of instructions for cellular functions that are expressed through biochemical processes. Cellular DNA, however, is also organized as a complex hierarchical structure with a mosaic of mechanical features, and a growing body of evidence is now emerging to imply that these mechanical features are connected to genetic function. Mechanical tension, for instance, which must be felt by DNA within the heavily constrained and continually fluctuating cellular environment, can affect a number of regulatory processes implicating a role for biomechanics in gene expression complementary to that of biochemical regulation. In this article, we review evidence for such mechanical pathways of genetic regulation.

## 1. Introduction

Critical to the coordinated functioning and development of cells is the ability to process mechanical signals and stimuli. A failure to respond appropriately to mechanical stress can have dire consequences that range from cellular apoptosis to malignant features such as cancer [1]. Cells are capable of detecting external mechanical stimulation by a variety of signal-transduction mechanisms. Typical examples are the activation of mechanosensitive ion channels, protein tyrosine kinases, small and large G proteins, and other signalling molecules within the cellular membrane [2,3]. These transduction elements convert mechanical forces acting on the surface of the cell into chemical signals that trigger an internal cellular response, which, at times, effectively result in force-dependent changes in gene expression further downstream of the signalling cascade. For instance, mechanical stimulation of the cell membrane can cause transcription factors like nuclear factor κB to translocate from the cytoplasm to the nucleus [4] and can induce mitogen-activated protein kinase cascades that activate various transcription factors [5].

**Figure 1.**
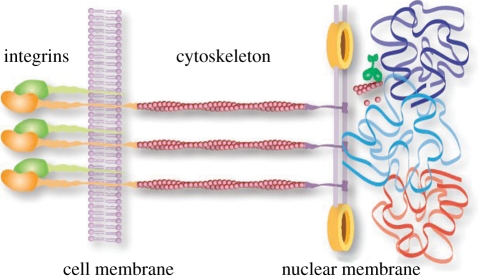
A network of mechanical connections have been identified within eukaryotic cells that could, in principle, transmit mechanical signals from receptors at the cell membrane, through the cytoskeletal architecture, past the nucleus and into the chromosome. Moreover, within the nucleus the DNA finds itself tightly packaged and operating within a bustling environment driven by gene expression and molecular motor activity.

It is not always necessary, however, to immediately convert a mechanical stimulus into a chemical signal right at the cell membrane. Certain membrane proteins, such as integrins and cadherins, can physically couple the extracellular matrix to the actin cytoskeleton, which in turn links to the nucleus [6], providing a route for the mechanical stimuli to propagate deep within a cell. These mechano-transduction pathways give rise to structural changes within the cell's interior, which deform the nucleus and, in turn, affect the nuclear lamina—a component that acts to preserve the shape and mechanical stability of the nucleus. The nuclear lamina is composed of fibrous proteins, known as nuclear lamins, that bind to DNA and chromatin [7] and are already known to play a role in transcription, replication and chromatin organization [8,9]. At each stage of the cellular architecture, from the cell membrane to the nuclear core, an interconnected network of physical structures is present that could, in principle, allow mechanical signals to regulate gene expression without a biochemical intermediary (figure 1).

While pathways in which a mechanical stimulus is converted early on into a chemical signal can be readily studied by standard methods of molecular biology, purely mechanical signalling is not nearly as amenable to experimental investigation because the tools for applying and measuring intracellular forces are sorely lacking. And although no purely mechanical pathway that directly regulates genetic function without a biochemical intermediate has yet been identified, we are now beginning to understand how internal forces applied to the DNA, resulting from the transduction of extracellular forces directly to the nucleus, or those stemming from cellular activity and repeated interaction with the chromosome, can affect genetic function. For instance, in both prokaryotes and eukaryotes, torsional stress induced by the procession of RNA polymerases can dynamically generate supercoils that directly influence activity farther downstream along the genome [10–12]. *In vitro* experiments have shown that it can take up to 20 pN of force to halt the procession of RNA polymerases [13], nucleosomes reversibly detach from DNA at around 50 pN [14], polymerases apply approximately 12 pN of force to initiate DNA strand separation [15], stiff microtubules can exert 47 pN of force on the chromosome during mitosis [16], and so on, which goes to show that much of the regulatory machinery is capable of generating significant forces, readily capable of bending, stretching and twisting both DNA and chromatin, and that these forces can have marked effects on cellular functions. However, it is still unclear how much tension is actually present along the chromosome of living cells during normal cellular operation or during a response to external stimulus.

A prescient indication of the importance of the cellular environment on genetic function came in the early Eighties when Arthur Kornberg realized that, for DNA replication to proceed *in vitro*, it was necessary to mimic the crowding present in living cells [17], which is very substantial with densities of up to several hundred grams per litre [18]. After a frustrating decade of trying to replicate stretches of the *Escherichia coli* chromosome outside of the bacteria in ordinary solution, success was achieved only by adding the crowding agent propylene glycol to the mixture. This finding was a startling, yet persuasive, admonition that we cannot neglect the cellular environment when explaining genetic behaviour.

Perhaps, more remarkable than the extent to which the cell is crowded is the fact that the cellular interior is far from thermal equilibrium. In fact, the mesh-like structure of the cytoskeleton is driven by activities, such as the procession of molecular motors, like myosin and actin treadmilling, both of which continually consume adenosine triphosphate (ATP) [19] generating an active medium within the cell. Experiments have shown that nanoparticles passively embedded within such a network can experience jostling from environmental fluctuations of more than 100 times those of thermal fluctuations alone [20]. These findings show that the cellular environment is much more dynamic than previously assumed, with potentially far-reaching consequences as to how cellular functions can be driven not only by thermal fluctuations or direct molecular motor action, but perhaps also by active mechanical fluctuations within the cell.

For more than half a century, it has been debated whether the nucleus contains a macromolecular scaffolding similar to that of the cytoplasm [21] and the subject, to this day, remains controversial. It is only in recent years that a polymeric form of actin has been found within the nucleus [22–24]. Likewise, the discovery of a nuclear relative of myosin, nuclear myosin I, was reported and shown to be a key player in nuclear function [25]. Furthermore, ATP-driven chromatin remodellers like the chromatin structure remodelling complex have been shown to exert forces comparable with those of more conventional molecular motors [26]. These new findings make it quite probable, although still unconfirmed, that the chromosome could experience similar non-equilibrium effects within the nuclear interior like those uncovered within the cytoplasm, which would have significant implications for overall gene expression.

This review aims to highlight recent evidence suggesting that forces, static or dynamically fluctuating, that act on the DNA backbone can directly alter gene expression. In the following section, §2, we will focus on discussing the effects that mechanical tension in the DNA can have in the local operation of DNA-binding proteins, such as on the assembly and activity of a variety of transcription factors and restriction enzymes. In §3, we will discuss long-range tension-induced cooperative effects like regulation via protein-mediated DNA looping and the mechanical operation of type II restriction endonucleases. In §4, we briefly review how mechanical tension, from forces generated along the nuclear DNA, may participate in the functioning of the chromosome. The variety of this set of examples illustrates the need to incorporate DNA mechanics into our evolving understanding of genetic regulation. In §5, we conclude with a discussion of recent efforts at and prospects for performing force spectroscopy within living cells to directly measure the static and dynamic forces and fluctuations that act on DNA *in vivo*. We hope that this review will help stimulate a nascent interest in uncovering novel mechanical pathways to gene regulation that rely upon tension within the DNA.

## 2. Localized protein-binding and aggregation

There is much evidence to suggest that transcription factor binding and unbinding is tension-dependent, although the biological implications of these findings are still unknown [27]. Single-molecule stretching experiments are able to observe a change in the elasticity of the DNA as transcription factors associate and/or dissociate from the DNA strand. Tension-dependent effects have been observed on a variety of transcription factors, such as the integration host factor [28] and heat unstable nucleoid protein (HU) [29], both important nucleoid associated proteins in bacteria, as well as a variety of non-specific DNA-bending proteins like HMGB1 and NHP6A [30]. In fact, it is the non-specific proteins, as these molecules are only weakly bound to the DNA, which should be the most sensitive to tension-dependent mechanical signals [31].

**Figure 2.**
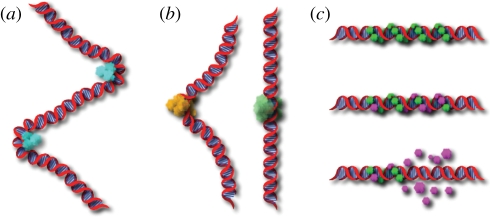
(*a*) Proteins bound to DNA may generate twist and tension along the molecule. One result may be an increased rate of aggregation driven by a need to reduce the deformational cost in energy associated with their binding. (*b*) Certain restriction endonucleases like EcoRI (yellow) must first bend the DNA before they can cut and display an activity level dependent upon tension in the molecule. Others, like BamHI (green), cause little distortion to the DNA and show little sensitivity to tension. (*c*) Disassembly of nucleoprotein filaments is a crucial step in recombination and is strongly tension-dependent. The filaments are stable as long as RAD51 proteins are bound to ATP (green). The RAD51 monomers, which have hydrolysed ATP (purple), dissociate from the filament once a terminal monomer hydrolyses ATP. Dissociation continues up until the point at which the next RAD51 monomer is found bound to ATP.

Once in contact with the DNA, the activity of transcription factors or other proteins is in some instances dependent upon tension along the DNA as well. For instance, when restriction endonucleases bind to their target sequences, they sometimes induce a significant conformational change in the DNA structure (figure 2*b*). EcoRV, as an example, is known to generate a kink with a bend angle of around 50°. Van den Broek *et al*. [32] showed that the rate of cleavage for EcoRV is greatly reduced when tension is applied to the substrate DNA. This is in contrast with the enzyme BamHI, which has little effect on the conformation of the DNA, and showed no tension-dependence in its activity.

A surprising example of how stored mechanical tension can affect protein activity is provided by RAD51 proteins that polymerize into filaments around ssDNA and act as catalysts for homologous recombination. The filaments assemble and help locate a homologous segment of dsDNA, the DNA molecules exchange strands of genetic material, the filaments disassemble and two homologous dsDNA molecules are formed. Van Mameren *et al*. [33] recently showed that the disassembly of these nucleoprotein filaments, which is an important step in the process of recombination, critically depends upon a release of tension in the filament. The disassembly process halts when tension is applied to the DNA, stabilizing the binding of the RAD51 protein, and re-commences once the tension is released. In fact, since RAD51 forms helical filaments in the DNA that can hold a significant amount of tension, the authors postulated that this stored mechanical energy might power the disassembly process (figure 2*c*).

A variety of transcription factors are also known to display a significant level of cooperativity that, along with the interplay between enhancers and repressors, enables a high degree of multivariant control over genetic function. For instance, this holds true for all three classes of RNA polymerases; specific transcription factors have been shown to assemble at the promoter into multiprotein–DNA complexes as a precondition for transcription initiation. The assembly of multiple proteins into a larger functional unit proves to be a common theme in gene regulation, as the cooperative binding of proteins to DNA allows for a sensitive response to small changes in protein concentration and the implementation of more complex control schemes.

The assembly of such multiprotein complexes is rather slow, as the kinetics is limited by the diffusion of the complex constituents at often very low concentrations. Elastic stress in a biopolymer, however, can propagate over a long range in a fraction of time. Propagation of such a mechanical signal is limited only by the fundamental relaxation time of the carrier polymer, which can be quite short even for micrometre-sized stretches of DNA [34]. Tension, therefore, could serve as an efficient long-range signal, which in turn alters the rate of transcription complex formation or organizes protein spacing along the DNA. Support for such a notion comes from the theoretical work of Rudnick & Bruinsma [35], who have shown that tension along the DNA strand can facilitate the cooperative binding of DNA as two DNA-bending proteins will tend to bind next to each other in an effort to minimize the overall distortion of the DNA molecule (figure 2*a*). Koslover & Spakowitz [36] then went on to extend this theoretical argument by showing that rotational twist in the DNA can play a role, complementary to that of tension, in aggregating proteins bound to the DNA.

## 3. Tension-dependent control of distant, cooperative processes

Proteins that bind far from the promoter they regulate can be brought near the initiation region for transcriptional regulation by looping the intervening DNA. This process of DNA looping is quite common in prokaryotes, being present in the *ara*, *gal, deo* and *lac* operons in *E. coli* [37] as well as the lysogenic/lytic switch in phage λ [38], and is ubiquitous throughout eukaryotes where it allows distal enhancers, silencers and mediators to affect transcriptional regulation [37,39].

DNA is tightly packed within the cell, particularly in eukaryotes. This dense cellular environment provides a complex micromechanical context in which the distantly bound protein has to find its counterpart at the promoter, and a variety of strategies are employed by the genome to either enhance or impede this process. Supercoiling, for instance, is a global mechanical feature for regulating gene expression and is caused by a linking number deficit of the DNA within a specific topological domain of the DNA. This in turn leads to the local formation of plectonemes in the DNA to relieve unwanted torsional stress [40]. This twist in the DNA is necessary, for instance, to bring the spatially distant *gal* operators of the *gal* operon in *E. coli* into the correct orientation with respect to each other, thus facilitating the closure of the repressor loop [41,42].

Like twist, tension in the substrate DNA can be critical to this sort of long-range regulatory function. DNA loop formation is driven by thermal fluctuations and intracellular interactions that randomly bend and twist the DNA. When two binding sites come in close proximity to one another, a regulatory protein may form a bridge between the operators to generate a loop in the intervening DNA. The force associated with thermal fluctuations, needed to form such a loop, can be estimated from the persistence length of the DNA at around 0.1 pN—only a fraction of the scale of forces exerted on the DNA during normal cell functioning, like those discussed in §1. It was, therefore, predicted that forces as small as a few hundred femtonewtons could supersede the thermal fluctuations and easily suppress the rate of formation of protein-mediated DNA loops [43,44], effectively preventing all loop formation and, in turn, dramatically altering transcription levels.

**Figure 3.**
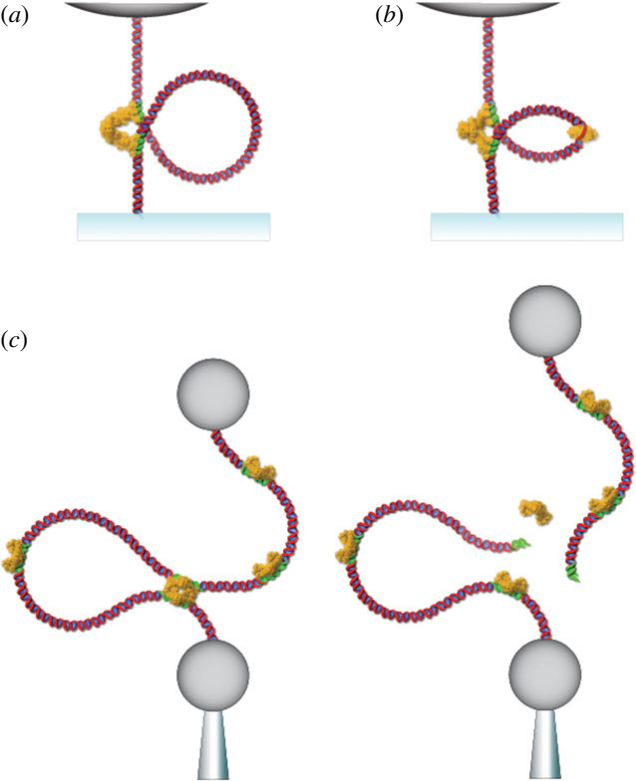
(*a*) LacI-mediated DNA loops form when distant operator sites (green) along the DNA come in close enough proximity that they may be bridged by an intermediary LacI protein. The formation rate of such loops is acutely sensitive to both femtonewton tensions and fluctuations along the DNA. (*b*) GalR-mediated DNA loops are assisted by HU proteins that increase the flexibility of the intervening DNA allowing loop lengths of below a persistence length to form. This regulatory mechanism has been shown to be quite sensitive to both twist and tension along the DNA. (*c*) Type II restriction endonucleases, like Sau3AI, cut when distant restriction sites encounter an active enzyme and display a strong suppression of activity at tensions of below a piconewton.

Recent experiments have probed the effect of mechanical tension on protein-mediated DNA looping by observing the formation and breakdown of DNA loops formed between two *lac* operator sites borrowed from the bacterial chromosome and bridged by tetrameric LacI protein (figure 3*a*). Chen *et al*. [45] have shown that protein-mediated DNA loops, *in vitro*, can be suppressed by applying only a few hundred femtonewtons of force to the substrate DNA. This result would translate *in vivo* to a complete suppression of the repressive effects generated by the loop causing the cell to continuously express the set of genes controlled by this operon. It should be noted that the loops, once formed, are hard to disrupt—their lifetime is essentially unaffected by forces on the sub-piconewton scale. To disrupt the lifetime of the DNA loops, it would require the application of a force almost two orders of magnitude greater than that necessary to affect the loop association rate. That is, the force would need to be comparable with the enthalpic cost of tearing a protein from its binding site (i.e. tens of piconewtons as opposed to tenths of a piconewton).

In a separate experiment, Chen *et al*. [46] found that by applying a fluctuating level of tension to the DNA they could greatly enhance the rate of loop formation. The experiment was meant to simulate the fluctuating micromechanical environment of the cellular interior, where fluctuating forces arise from a wide range of intracellular processes. The introduced fluctuations were formally equivalent to increasing the effective temperature of the system and it was found that the loop formation rate could be more than doubled by adding an effective temperature of only 10 per cent of the thermal background. This rate enhancement, owing to force fluctuations, might explain why DNA loops result in a several 100-fold level of repression *in vivo* [47] despite the observation of equal lifetimes in the looped and unloooped states *in vitro*. Moreover, the sensitivity of the loop formation rate to the additive fluctuations was shown to be independent of the baseline static tension in the substrate DNA. This led the authors to suggest that schemes which employ mechanical tension as a regulatory switch can be surprisingly robust even in a mechanically noisy environment.

A similar phenomenon to what was observed in the *lac* system was witnessed by Gemmen *et al*. [48,49] in a novel set of type II restriction enzymes that cleave DNA efficiently only if there are multiple recognition sites along the DNA (figure 3*c*). The activity of these enzymes suggests that the complex simultaneously binds at the two sites forming a loop in the intervening DNA. Such a behaviour is thought to protect against unwanted cleavage should a single site in the host's genome accidently become unmethylated [50]. Gemmen and co-workers studied 15 two-site enzymes by observing cleavage events under varying levels of tension. They found the activity of all the two-site enzymes to be completely inhibited by a mere tension of 5 pN. A detailed study of one of the enzymes, Sau3AI, showed an exponential decrease in activity as a function of tension resulting in a 10-fold suppression of activity at less than 1 pN. While the *lac* results discussed earlier were in excellent agreement with theory [43,44], here, a 10-fold level of suppression was predicted to occur at around 0.1 pN. Gemmen and co-workers speculate that the discrepancy might result from the formation of many small, classically unfavourable DNA loops, arising from protein-induced or spontaneous DNA bending, requiring additional tension to inhibit.

In certain regulatory systems, DNA loop formation may be assisted by additional proteins that help increase the flexibility of a rather stiff segment of DNA. For instance, transcriptional repression of the previously discussed metabolic *gal* operon, by the repressor protein GalR, involves the formation of a protein-mediated DNA loop of approximately 40 nm, slightly below the persistence length of dsDNA (figure 3*b*). DNA loop formation in this system requires the assistance of the nucleoid-association protein HU, which is thought to bind and to denature a portion of the DNA between the distant operator sites [42]. Since ssDNA is much more flexible than dsDNA, the overall flexibility of the DNA is increased and the formation of short DNA loops is significantly facilitated. In the same experiment, Lia *et al*. [42] showed that negative supercoils in the DNA, generated under linear tension of the order of 1 pN, massively facilitated the formation of GalR/HU-mediated DNA loops. Moreover, as they increased the applied tension, they found that the probability of looping decreased, however, not as dramatically as was found in the LacI experiments discussed above. This result is most likely owing to the increased flexibility, or reduced effective persistence length, of the DNA in the GalR/HU loops, which in turn increases the characteristic force scale of the thermal fluctuations of the DNA.

## 4. Mechanical effects and the chromosome

The ability of a given stretch of DNA to wrap itself around a nucleosome is strongly dependent upon the sequence [51,52] with a range of affinities that span three orders of magnitude [53]. There is evidence to suggest that the genome uses this sequence dependence to preferentially govern the distribution of nucleosomes as a method for controlling the access of regulatory proteins to particular binding sites [54]. Tension may play a complementary role to sequence as it may influence the binding of nucleosomes to the DNA and, therefore, has the potential to be an important determinant of nucleosomal positioning.

Single-molecule experiments have already shown that individual histone octomers reversibly detach from DNA under piconewton tensions [14]. Moreover, *in vitro* nucleosome disruption experiments, performed in ATP-rich *Xenopus* extracts, have shown that 2 pN of force can completely disassemble a chromatin fibre [55]. Oddly enough, an *in vivo* measurement performed in yeast cells indicated that nucleosome disruption could occur at tensions as small as 0.2 pN [56], which is an order of magnitude less than what was witnessed in the *in vitro* experiments with *Xenopus* extracts.

**Figure 4.**
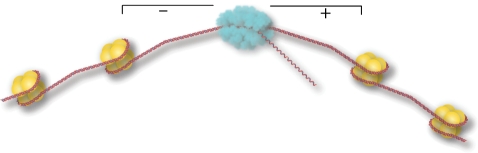
During the transcriptional activity of RNA polymerase, additional torque is generated from the processive motion of the polymerase. This torque results in positive supercoils along the template DNA downstream of the polymerase that may help unwrap nucleosomes, clearing the DNA for transcription. Likewise, negative supercoiling upstream of the polymerase might aid in the reassembly of nucleosomes with the template DNA.

Another way in which mechanical stress can affect nuclear DNA is by acting as an active messenger of genetic activity. For instance, experiments have shown that transient mechanical stresses induced by molecular motors can propagate through chromatin fibre and cause local alterations to DNA. Kouzine *et al*. [57] as an example, originally showed that transcriptionally generated torque is capable of melting sequences hundreds of basepairs upstream of an active promoter *in vitro* (figure 4). In a follow-up to this work, the same group witnessed transcriptionally generated supercoiling, but this time *in vivo* using psoralen, a compound whose binding affinity to DNA is dependent upon the helical tension in the DNA, as an indicator [12]. The resulting superhelical tension caused the structure of the DNA to deviate from the traditional B-form along sequences located upstream of the promoter. This mechanical signal caused one of the affected sequences, the far upstream element (FUSE), to recruit two regulatory proteins essential to its function showing that mechanical signals, resulting from structural features of the chromosome, can participate in gene regulation.

On a more global scale, the spatial organization of DNA within a cell gives rise to additional mechanisms that have the potential for direct mechanoregulation. There is a growing body of evidence for a division of the bacterial chromosome into supercoiled domains [58,59] in which helical tension regulates DNA transcription [60,61]. A similar statement about the eukaryotic chromosome is more contentious [62]; however, the chromosome seems to be organized in such a way as to take advantage of and control DNA tension [63].

Moreover, there is much evidence to suggest that cells can control gene expression by altering the spatial organization of the nucleus, for instance, by making certain regions of the chromosome inaccessible [64,65], to orchestrate the expression or inhibition of large clusters of genes. In this way, the chromosome actively participates in the control of its expression through its own packaging—a feature that is strongly dependent upon the mechanical properties of chromatin.

Inside the cell, for instance, the chromosome finds itself effectively caged within the nucleus. This crowding can give rise to internally generated mechanical forces that may drive rearrangements of the chromosome. In eukaryotic cells, an extension of the chromosomal fibre can result from a variety of processes, such as histone modification, elimination or the loss of non-histone architectural elements. Under such conditions, the DNA would play a governing role beyond its information content, rather via its mechanical features. Kleckner *et al*. [63] noted that during periods of cellular expansion, chromosomes tend to be distended and stiff while, during periods of contraction, the chromosomes become flaccid and relaxed. These periods of tensional modulation, during the cell cycle, were postulated to be a mechanical source for regulating cellular activity. Recently, *in vivo* measurements have been made of the tension exerted by the microtubule-based spindles, present during cell division, upon a length of chromatin [56]. The DNA/chromatin was extended within the cell with a maximum force of approximately 0.2 pN showing that significant forces may act upon the chromosome during various stages of the cell's life cycle.

## 5. Outlook for exploring the mechanics of gene regulation

Most of our ideas on how DNA mechanics might regulate gene expression arise from *in vitro* experiments that study the response of extracted or artificially synthesized cellular components. This approach has clearly established the plausibility and even probability of direct mechanical pathways to regulate gene expression that do not require an intermediate conversion into a chemical signal at the cell membrane; however, at present, no single mechanism that is actually used by living cells has been conclusively demonstrated. To do so will require an extension of present-day techniques to both observe and probe mechanical function within the complicated cellular interior.

**Figure 5.**
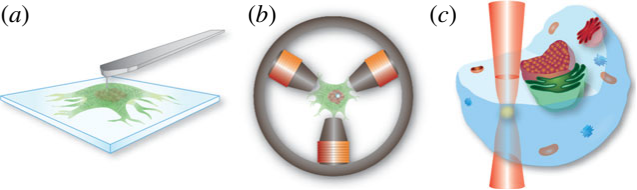
(*a*) Silicon and carbon nanoprobes, attached to micromanipulators or modified atomic force microscope tips, can be directly inserted into the cytoplasm and, in some cases, through to the nucleus while causing only minimal interference with cellular activity. (*b*) Micromagnetic tweezers have been used to study the active microrheology of magnetic nanoparticles injected into the nucleus of eukaryotic cells. (*c*) Optical tweezers can be used to manipulate refractile bodies within cells, such as lipid vesicles, and have been used to measure the transport properties of various molecular motors *in vivo*.

There are many challenges to perform force spectroscopy measurements within the *in vivo* world [66,67], but, it is a direction in which many single-molecule biophysical techniques are moving, albeit incrementally and at an agonizingly slow pace. One early and very direct method of applying mechanical forces to cellular components *in vivo* is through the insertion of microneedles into living cells (figure 5*a*). Skibbens & Salmon [68] used such an approach to test if kinetochores respond to tension during various stages of cell division in newt epithelial cells. They were able to stretch the chromosomal arms directly with microneedles that were punched through the cellular membrane, and thereby apply an external force to the spindle. Their results showed that tension controls the direction of kinetochore movement and the associated assembly and disassembly of the microtubules, which act to position the centromere within the spindle. These microneedles, generated by heating and pulling on a segment of glass tubing and then finely tapering the end, however, were rather large on a cellular scale. In recent years, much smaller diameter microneedles have been fashioned using modern nanofabrication techniques, which translate to less damage to the cells under investigation, and allows for more control over the positioning and manipulation of the tips. For instance, modified Si atomic force microscope tips, created by focused ion beam etching, with a diameter of 200–300 nm have been shown to penetrate both the cell membrane as well as the nuclear membrane [69]. Even more recent is the development of nanoneedles that are created from carbon nanotubes of merely 30–40 nm diameters. They can both interact with internal cellular components as well as deliver a variety of nanoparticle payloads to the cellular/nuclear interior [70]. Micro- and nanoneedles remain, however, a fairly invasive method to probe intracellular mechanics, with not insubstantial collateral cellular damage.

Another option for *in vivo* cellular manipulation is through optical or magnetic tweezers. Optical tweezers have unearthed a wealth of mechano-molecular information in *in vitro* assays of biological function. They are, however, less adept at exploring the intracellular milieu because they lack specificity and exert forces only based on a difference in refractive index, something that cannot easily be tailored within the cell using ordinary molecular biology techniques. One solution to this problem, which has recently been employed to study active transport within a cell, was to trap lipid droplets that are more readily trapped than other cellular components [71] (figure 5*b*). However, the laser intensities needed for these studies were high enough to cause significant local heating and possible photodamage to the cell, a limitation that will probably be a limiting factor in the development of other intracellular handles for nanomechanical studies with optical tweezers.

Magnetic tweezers, on the other hand, are a promising option for *in vivo* studies in that most cellular components show very little magnetic susceptibility and therefore high specificity can be achieved by introducing functionalized superparamagnetic particles into the cell. A drawback of magnetic tweezers, though, is that complete three-dimensional control, like that afforded by optical tweezers, is difficult to realize. Microfabricated magnetic tweezers, which allowed for real two-dimensional control, were recently used to manipulate microinjected superparamagnetic beads inside the nucleus of a HeLa cell, to actively measure the nuclear elasticity and viscosity [72] (figure 5*c*). However, to manipulate the nanoprobes with sufficient force required the use of rather large 500 nm beads, which hardly moved within the nucleus and may well be disruptive to nuclear function. Nonetheless, this technique may lend itself to actively interacting with chromatin by conjugating the nanoparticles to specific histones, to directly exert mechanical forces on the chromosome and to observe their concomitant effects on gene expression.

While much effort is being put in to understand how the genetic code operates within the genome, there is a growing realization that the mechanics of transcriptionally active DNA may be responsible for a wealth of regulatory function in its own right. Exploring this ‘mechanome’ [73] will require the development of new tools and techniques for directly and specifically measuring and exerting forces on the DNA within living cells. Despite the hurdles, technical advances are moving us steadily closer to performing single-molecule measurements within living cells, laying the foundation for a new paradigm of how we interact with and explore the cellular world.
